# Supplementary material for: Super-resolution imaging in whole cells and tissues via DNA-PAINT on a spinning disk confocal with optical photon reassignment
Source: Nat Commun. 2025 May 29;16:4991. doi: 10.1038/s41467-025-60263-w (PMC12122864; doi:10.1038/s41467-025-60263-w)
Supplement: Supplementary file 2 — Reporting Summary [file 41467_2025_60263_MOESM2_ESM.pdf]

nature portfolio

Corresponding author(s): Dr. Sabrina Simoncelli

Last updated by author(s): May 8, 2025

Reporting Summary

Nature Portfolio wishes to improve the reproducibility of the work that we publish. This form provides structure for consistency and transparency in reporting. For further information on Nature Portfolio policies, see our Editorial Policies and the Editorial Policy Checklist.

Statistics

For all statistical analyses, confirm that the following items are present in the figure legend, table legend, main text, or Methods section.

n/a	Confirmed	
☐	☒	The exact sample size (*n*) for each experimental group/condition, given as a discrete number and unit of measurement
☐	☒	A statement on whether measurements were taken from distinct samples or whether the same sample was measured repeatedly
☒	☐	The statistical test(s) used AND whether they are one- or two-sided<br>*Only common tests should be described solely by name; describe more complex techniques in the Methods section.*
☒	☐	A description of all covariates tested
☒	☐	A description of any assumptions or corrections, such as tests of normality and adjustment for multiple comparisons
☐	☒	A full description of the statistical parameters including central tendency (e.g. means) or other basic estimates (e.g. regression coefficient) AND variation (e.g. standard deviation) or associated estimates of uncertainty (e.g. confidence intervals)
☒	☐	For null hypothesis testing, the test statistic (e.g. F, t, r) with confidence intervals, effect sizes, degrees of freedom and P value noted<br>*Give P values as exact values whenever suitable.*
☒	☐	For Bayesian analysis, information on the choice of priors and Markov chain Monte Carlo settings
☒	☐	For hierarchical and complex designs, identification of the appropriate level for tests and full reporting of outcomes
☒	☐	Estimates of effect sizes (e.g. Cohen's *d*, Pearson's *r*), indicating how they were calculated

Our web collection on statistics for biologists contains articles on many of the points above.

Software and code

Policy information about availability of computer code

Data collection	SDC and SDC-OPR raw images were acquired using NIS-Elements AR<br>TIR raw images were acquired using µManager (v.2.0.0), available at https://micro-manager.org
Data analysis	Deconvolution of the SDC-OPR images was performed using NIS-Elements AR (v.5.42.06)<br>Raw fluorescence videos were processed for super-resolution reconstruction using the Picasso software package (v.0.7.0), latest version accessible at https://github.com/jungmannlab/picasso and custom code<br>qPAINT analysis was performed on MATLAB (v.2022a), with code accessible at https://github.com/Simoncelli-lab/qPAINT_pipeline<br>3D super-resolution imaging was done using the 2D cspline PSF experimental model from SMAP software https://github.com/jries/SMAP

For manuscripts utilizing custom algorithms or software that are central to the research but not yet described in published literature, software must be made available to editors and reviewers. We strongly encourage code deposition in a community repository (e.g. GitHub). See the Nature Portfolio guidelines for submitting code & software for further information.

Data

Policy information about availability of data

All manuscripts must include a data availability statement. This statement should provide the following information, where applicable:

- Accession codes, unique identifiers, or web links for publicly available datasets
- A description of any restrictions on data availability
- For clinical datasets or third party data, please ensure that the statement adheres to our policy

Source data are provided with this paper. Single-molecule localization data generated in this study have been deposited in the Figshare repository, https://doi.org/10.6084/m9.figshare.28741373.

Research involving human participants, their data, or biological material

Policy information about studies with human participants or human data. See also policy information about sex, gender (identity/presentation), and sexual orientation and race, ethnicity and racism.

Reporting on sex and gender	No research involved reporting on sex or gender.
Reporting on race, ethnicity, or other socially relevant groupings	No research involved reporting on race, ethnicity, or other socially relevant groupings.
Population characteristics	No research involved reporting on population characteristics.
Recruitment	No research involved recruitment.
Ethics oversight	n/a

Note that full information on the approval of the study protocol must also be provided in the manuscript.

Field-specific reporting

Please select the one below that is the best fit for your research. If you are not sure, read the appropriate sections before making your selection.

☒ Life sciences ☐ Behavioural & social sciences ☐ Ecological, evolutionary & environmental sciences

For a reference copy of the document with all sections, see nature.com/documents/nr-reporting-summary-flat.pdf

Life sciences study design

All studies must disclose on these points even when the disclosure is negative.

Sample size	No sample size calculation was performed. In general, sample sizes were kept as big as practically possible with the described microscopy technique and type of illumination.
Data exclusions	No data was excluded.
Replication	All replications (3 for DNA-origami samples, Nuclear Pore Complex and Drosophila retina samples; 3 for microtubules samples and 2 for mitochondria samples) were successful.
Randomization	No grouping of experiments or samples was performed.
Blinding	No grouping of experiments or samples was performed.

Reporting for specific materials, systems and methods

We require information from authors about some types of materials, experimental systems and methods used in many studies. Here, indicate whether each material, system or method listed is relevant to your study. If you are not sure if a list item applies to your research, read the appropriate section before selecting a response.

Materials & experimental systems

n/a	Involved in the study
☐	☒ Antibodies
☐	☒ Eukaryotic cell lines
☒	☐ Palaeontology and archaeology
☐	☒ Animals and other organisms
☒	☐ Clinical data
☒	☐ Dual use research of concern
☒	☐ Plants

Methods

n/a	Involved in the study
☒	☐ ChIP-seq
☒	☐ Flow cytometry
☒	☐ MRI-based neuroimaging

Antibodies

Antibodies used	1. Rat monoclonal (YL1/2) anti-alpha tubulin, Thermo Fisher Scientific (Cat#MA1-80017), 5 µg/ml<br>2. Alpaca sdAb (1H1) anti-GFP coupled with DNA, Massive Photonics (Cat#MASSIVE-TAG-Q-FAST anti-GFP - Cy3B), 20 nM<br>3. Alpaca sdAb (1H1) anti-GFP coupled with custom DNA (sdAB-5'-TCCTCCTCCTCCT-3'), Massive Photonics (custom product), 20 nM<br>4. TOM20 antibody (Ab186735, abcam), 1:200 dilution<br>5. Anti-rabbit IgG (Massive Photonics, Massive-sdAB-FAST 2-Plex, Secondary sdAB F2), 25 nM<br>6. Anti-TCRζ (6B10.2, 644102, BioLegend), 10 ug/ml
Validation	1, 4 and 6: Verified by western blot and/or immunofluorescence by manufacturer to ensure that it binds to antigen stated.<br>2, 3 and 5: Specificity test by NanoTag

Eukaryotic cell lines

Policy information about cell lines and Sex and Gender in Research

Cell line source(s)	HeLa Kyoto with endogenous Nup107 tagged with mEGFP (CLS Cell Lines Service GmbH).<br>U2-OS CRISPR mEGFP-Nup96 (Cytion)<br>Jurkat E6.1 T cells
Authentication	Cell lines were not authenticated.
Mycoplasma contamination	All cell lines have been tested negative for mycoplasma contamination
Commonly misidentified lines (See ICLAC register)	No commonly misidentified cell lines were used.

Animals and other research organisms

Policy information about studies involving animals; ARRIVE guidelines recommended for reporting animal research, and Sex and Gender in Research

Laboratory animals	Drosophila melanogaster was used in this study. Modified strains BDSC:60584 and BSDC:98343 were obtained from the Bloomington Drosophila Stock Center. 3rd instar larvae and pupae were used in this study.
Wild animals	No wild animals were involved in this study.
Reporting on sex	Both male and female animals were used. Our data was not sex disaggregated. We do not anticipate that biological sex would impact the results of this study.
Field-collected samples	No field-collected samples.
Ethics oversight	No ethical approval was required for this study as it utilized Drosophila melanogaster and cultured cell lines.

Note that full information on the approval of the study protocol must also be provided in the manuscript.

Plants

Seed stocks	No plants were involved in this research.
Novel plant genotypes	No plants were involved in this research.
Authentication	No plants were involved in this research.
